# Chronic Cadmium Exposure Induces Impaired Olfactory Learning and Altered Brain Gene Expression in Honey Bees (*Apis mellifera*)

**DOI:** 10.3390/insects13110988

**Published:** 2022-10-27

**Authors:** Zhiguo Li, Yuanmei Qiu, Jing Li, Kunlin Wan, Hongyi Nie, Songkun Su

**Affiliations:** College of Animal Sciences (College of Bee Science), Fujian Agriculture and Forestry University, Fuzhou 350002, China

**Keywords:** *Apis mellifera*, proboscis extension response, olfactory learning, cadmium, transcriptome analysis

## Abstract

**Simple Summary:**

The honey bee (*Apis mellifera*) is kept all over the world and plays a dominant role in the pollination of crops. Honey bees may be exposed to different levels of cadmium through the collection of contaminated nectar during their foraging activities. In this study, honey bees were chronically exposed to cadmium to investigate the effects of sublethal cadmium doses on the olfactory learning and brain gene expression profiles in honey bees. Honey bees exhibited significantly impaired olfactory learning performances after being chronically exposed to cadmium and had a significantly lower head weight in comparison with control bees. Furthermore, genes involved in oxidative stress response and odor sensing were dysregulated in the brain of cadmium-treated bees. These results suggest that cadmium exposure exerted oxidative stress and decreased gene expression levels of chemoreceptors in honey bees, which probably resulted in the impaired olfactory learning of honey bees.

**Abstract:**

The honey bee (*Apis mellifera*) plays vital ecological roles in the pollination of crops and the maintenance of ecological balance, and adult honey bees may be exposed to exogenous chemicals including heavy metals during their foraging activities. Cadmium (Cd) is regarded as a nonessential toxic metal and is readily accumulated in plants; honey bees can therefore acquire Cd through the collection of contaminated nectar. In the present study, honey bees were chronically exposed to Cd to investigate the effects of sublethal cadmium doses on the olfactory learning and brain gene expression profiles of honey bees. The results showed that Cd-treated bees exhibited significantly impaired olfactory learning performances in comparison with control bees. Moreover, the head weight was significantly lower in Cd-treated bees than in control bees after chronic exposure to Cd. Gene expression profiles between the Cd treatment and the control revealed that 79 genes were significantly differentially expressed. Genes encoding chemoreceptors and olfactory proteins were downregulated, whereas genes involved in response to oxidative stress were upregulated in Cd-treated bees. The results suggest that Cd exposure exerts oxidative stress in the brain of honey bees, and the dysregulated expression of genes encoding chemoreceptors, olfactory proteins, and cytochrome P450 enzymes is probably associated with impaired olfactory learning in honey bees.

## 1. Introduction

Environmental pollution by heavy metals is becoming an increasingly common problem around the world [1,2], and great attention has been paid to the adverse health risks of heavy metal exposure on humans and other organisms [3]. Cadmium (Cd) ranked seventh in toxicity among heavy metals according to the ATSDR [3]. The transfer of Cd from soil to agricultural plants occurs easily [4,5]; therefore, Cd is readily accumulated in plants and can enter the food chain straightforwardly, posing a risk to human and animal health [4,6].

Cd is regarded as a nonessential toxic metal, and it exists in soil, sedimentary rocks, and the lithosphere in the concentration range of 0.2–0.53 mg/kg [7]. Cd concentrations in agricultural soils rarely exceed 10 μg/L; however, anthropic activities, including mining, vehicle exhausts, the excessive use of phosphate fertilizer, and waste disposal have led to increased quantities of Cd released into the soil environment [7,8]. Elevated levels of Cd in soils and its transfer to the human body via the food chain cause health injury including skeletal damage and neurotoxic effects [9,10]. In addition, experimental evidence suggests that mice exhibited impaired cognitive abilities after chronic exposure to Cd at a concentration of 3 mg/L [11]. A higher exposure concentration of Cd contributed to growth limitation and fruit abortion in plants [12].

The honey bee (*Apis mellifera*) is kept all over the world and plays vital ecological roles in the pollination of crops and the maintenance of ecological balance [13]. Honey bees are a good model for assessing the ecological and health risks of toxic heavy metals on biota [14]. Honey bees are social insects, and worker bees of different ages perform different tasks. Young worker bees perform in-hive tasks, and old worker bees perform out-hive tasks, such as foraging [15]. Honey bee foragers may encounter various environmental factors including pesticides and heavy metals during their foraging activities [14]. Mounting evidence has suggested that forager bees exposed to exogenous chemicals exhibited behavioral and cognitive impairments that may lead to homing failure in forager bees and colony losses [16,17]. The olfactory proboscis extension response (PER) is a behavioral paradigm for evaluating the cognitive ability of honey bees under different conditions [18]. The olfactory learning behavior is closely related to the age of bees and has been shown to be impaired by biotic and abiotic factors [19,20,21].

Previous studies reported that the concentration of Cd in fruits from three different orchards exceeded the tolerance limit for Cd in fruits in Guangzhou, South China [5]. Honey bees can therefore acquire heavy metals through the collection of contaminated nectar, and heavy metal lead accumulated in the nectar of sunflowers decreased bee visit durations [22]. The intake of sucrose solution was decreased in foragers following joint exposure to Cd and copper (Cu), and joint Cd and Cu exposure delayed the development of honey bee larvae [23]. The olfactory learning of honey bees is an essential cognitive function and is required for effective foraging that plays vital roles in plant–honey bee interactions [24]. So far, most studies have found that honey bees are prone to environmental stressors including pesticides and heavy metals that affect the physiology and development of honey bees [23,25,26]. Thus, the honey bee has been regarded as a bioindicator of a polluted environment [27]. However, little is known about the effects of Cd exposure on olfactory learning in honey bees. We, therefore, investigated whether honey bees exposed to chronic Cd exhibited altered olfactory learning, and we further compared the patterns of brain gene expression between cadmium-exposed bees and control bees using RNA-seq analysis. Our findings provide the first evidence that the olfactory learning performances of honey bees can be impaired by chronic cadmium exposure. Furthermore, the dysregulated expression of genes encoding chemoreceptors, olfactory proteins, and cytochrome P450 enzymes was probably associated with Cd-impaired olfactory learning.

## 2. Materials and Methods

### 2.1. Honey Bees

Mature brood combs were removed from three different hives and placed in an incubator (34.5 °C, 70% RH). The newly emerged bees were collected and marked on the thorax using water-based paints within 24 h, and the paint-marked bees were then returned to the colony. The paint-marked bees were recovered from the colony after 5 days. A total of 30 same-aged bees were reared in each cage, which we have used previously [28], and eight cages of bees were prepared. The caged bees were placed in an incubator (30 °C, 70% RH) and subjected to Cd exposure.

### 2.2. Cadmium Exposure

A cadmium chloride (CdCl_2_) stock solution (25 μg/mL) was prepared by dissolving CdCl_2_ in sterilized water. The stock solution was diluted 100-fold with 30% sucrose solution (*w/v*). The cages of bees were randomly divided into two groups with each group consisting of four cages of bees. The bees in the treatment group were fed with 30% sucrose solution containing 0.25 μg/mL CdCl_2_, and the bees in the control group were fed with 30% sucrose solution. For each cage of honey bees, a 5 mL plastic syringe with the tip cut off was used as the feeder, and the amount of total sugar consumption was recorded directly from the syringe measure. All of the caged bees were fed ad libitum. Sugar consumption and the mortality of three cages of bees of each group were recorded during 18 days of Cd exposure. The sugar consumption per bee per day was calculated by dividing the amount of total sugar consumption by the number of remaining live bees each day. The survival rates were analyzed according to the number of remaining bees from each of the three control cages and the three treatment cages on the last day of the observation.

### 2.3. Olfactory Learning Experiments

Honey bees from each group were individually collected from the cage after 18 days of Cd exposure, and each bee was placed in a glass vial on ice to anesthetize the bees. After cold anesthesia, each immobilized bee was individually restrained in a customized tube with duct tape, leaving the head fixed and the proboscis moving freely. The harnessed bees were kept in an incubator (30 °C, 70% RH) for 2 h to recover from cold anesthesia before the training test of olfactory learning. The antennae of each bee were touched with a 50% sucrose solution (*w/v*), and the honey bees not showing proboscis extension were excluded from further behavior analysis. The training test of olfactory learning was adapted from Bitterman et al. [28], and odors A and B (A: 1-nonanol, B: hexanol) were presented in the experiment. Odor A (conditioned stimulus, CS +) was paired with a sugar-water reward (unconditioned stimulus, US), and odor B was paired with no reward (CS −). Each bee was exposed to the two odors according to the pseudorandom order (ABBAAB). One odor was used in each training test, and the test interval was 10 min.

Firstly, each harnessed bee was positioned in front of an olfactometer (kindly provided by M. Giurfa, University of Toulouse, France) and exposed to clean air for 15 s [29]. Odor A was then delivered for 4 s, and the bee was allowed to consume 50% sucrose solution (*w/v*) for 2 s after 2 s of odor A delivery. Finally, the bee was exposed to clean air for 20 s. An intact olfactory training test lasted 39 s. Odor B was delivered to the bee using the same training protocol, but without pairing with sucrose solution. The number of bees showing olfactory learning to odor A was recorded. The head weight of the honey bees from each group was measured after the training test.

### 2.4. RNA Isolation and RNA-Seq Library Preparation

A total of thirty honey bees were used for RNA isolation in each group, and the brain of each honey bee was dissected. Brains from ten honey bees were pooled into a single sample, and three pooled brain samples were used for RNA extraction in each group. The total RNA of each pooled brain sample was extracted with Trizol reagent (Invitrogen, CA, USA) following the manufacturer’s instructions. The RNA quality was evaluated with both Agilent 2100 Bioanalyzer (Agilent Technologies, Santa Clara, CA, USA) and agarose gel assay. Oligo(dT) beads were used to enrich mRNA from total RNA. The mRNA was fragmented by fragmentation buffer, followed by cDNA synthesis with random primers. DNA polymerase I, RNase H, dNTP, and buffer were used to synthesize the second strand of cDNA. After purification, the cDNA ends were repaired. The poly (A) was added to the cDNA before adapter ligation. The adapter-ligated cDNAs were size selected with agarose gel assay prior to PCR amplification. The cDNAs were sequenced using an Illumina HiSeq 2500 platform by Gene Denovo Biotechnology Co. (Guangzhou, China).

### 2.5. RNA Sequencing and Analysis

The raw reads were filtered by fastp (version 0.18.0) to remove reads containing adapters and low-quality reads [30]. The raw reads were further processed by Bowtie2 version 2.2.8 to remove rRNA-mapped reads [31]. The remaining paired-end clean reads were mapped to the *Apis mellifera* L. reference genome (Amel_HAv3.1) using HISAT2. 2.4 [32]. The mapped clean reads were assembled by StringTie v1.3.1, and the expression abundance of each transcript was calculated using the FPKM (fragments per kilobase of transcript per million fragments mapped) method. Analysis of the differentially expressed genes (DEGs) between the two groups of bees was performed by DESeq2 [33]. The Gene Ontology (GO) database was used to determine gene functional classification, and all DEGs were mapped to GO terms in the GO database [34]. Pathway enrichment analyses of DEGs were carried out based on the Kyoto Encyclopedia of Genes and Genomes (KEGG) database [35].

### 2.6. Quantitative Real-Time PCR

Nine differentially expressed genes were selected at random to validate the reliability of the RNA-seq results using quantitative real-time PCR. The brains of the honey bees of each group were dissected, and the brains of three honey bees were pooled into a single sample. Seven pooled brain samples were used for RNA extraction in each group. The total RNA was extracted from the brain samples of two groups of bees using the same method mentioned above. The first cDNA strand was synthesized from 1 mg of total RNA using the PrimeScript RT reagent Kit with gDNA Eraser (TaKaRa, Dalian, China) according to the manufacturer’s instructions. The qPCR reaction mixture (10 µL) contained 5 µL 2× SYBR Premix Ex Taq II (Tli RNaseH Plus), 0.2 µL of each PCR primer (10 µM) (Table S1) [36], 1 µL of diluted cDNA (1:3 dilution), and 3.6 µL of RNase free water. All reactions were run in triplicate in a Bio-Rad CFX 384 Real-time system. The reaction conditions were as follows: 95 °C for 30 s, followed by 40 cycles of 15 s at 95 °C and 30 s at 55 °C, followed by melting curve analysis. The relative expression levels of each target gene were analyzed using the 2^−∆∆CT^ method [37].

### 2.7. Statistics

The unpaired Student’s t-test was used to determine the statistical significance in survival rates, sugar consumption, head weight, and the relative gene expression level of nine target genes between control and cadmium-exposed bees. Statistical significance in olfactory learning performances was determined by Fisher’s exact test. For RNA-Seq data analysis, the transcripts with *p*-value < 0.05 and absolute fold change > 2 were considered differentially expressed transcripts between the two groups of bees. Significantly enriched GO terms and KEGG pathways related to DEGs were determined by a hypergeometric test.

## 3. Results

### 3.1. Syrup Consumption and Survival Analysis

**Figure 1:**
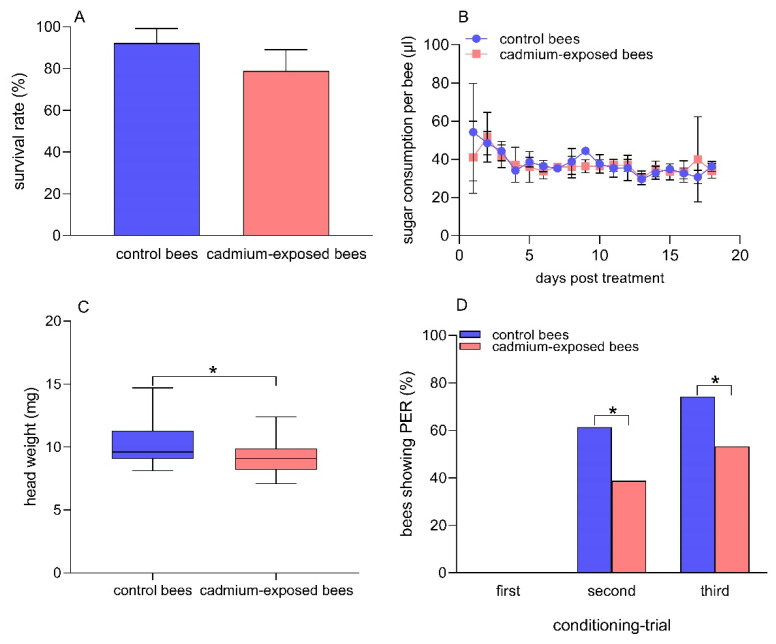
Comparison between cadmium-exposed and control bees in survival rate (**A**) sugar consummation per bee; (**B**) head weight; (**C**) Student’s t-test, n = 21 in both groups of bees, and the percentage of bees showing PER when presented with the CS +, 1-nonanol; (**D**) Fisher’s exact test, n = 70 for control bees, n = 62 for cadmium-exposed bees after chronic Cd exposure. (*) *p* < 0.05.

After 18 days of exposure to Cd, the mean survival rates of the control bees and the cadmium-exposed bees were 92.2% and 78.9%, respectively (Figure 1A). There was no significant difference in the mean survival rate between the two groups of bees (t = 1.9, df = 4, *p* = 0.13). The average sugar consumption per bee per day was 37.8 ± 6.3 µL and 37.0 ± 4.6 µL in the control bees and the cadmium-exposed bees for the 18 days of exposure to Cd, respectively (Figure 1B). There was no significant difference in the sugar consumption per bee between the two groups of bees (t= −0.42, df = 34, *p* = 0.18). The data suggested that 30% sucrose solution containing 0.25 μg/mL CdCl_2_ showed no significant repellent effects on honey bees, and the dose of CdCl_2_ used in our studies showed no acute toxic effects on the survival of honey bees.

### 3.2. Head Weight and Olfactory Learning Performances

The average head weight of the control bees and the cadmium-exposed bees was 10.4 mg and 9.4 mg, respectively (Figure 1C). The head weight was significantly lower in the cadmium-exposed bees than in the control bees after chronic exposure to Cd (t = 2.2, df = 40, *p* = 0.037). We observed that there was no PER triggered by the odor 1-nonanol (odor A, CS +) in either group of bees in the first learning test. The percentage of honey bees showing PER to 1-nonanol was 61.4% and 38.9% in the control bees and the cadmium-exposed bees in the second learning test, respectively. In addition, the percentage of honey bees showing PER to 1-nonanol was 74.3% and 53.2% in the control bees and the cadmium-exposed bees in the third learning test, respectively. The control bees showed significantly better olfactory learning abilities than chronic Cd-exposed bees in the second (Fisher’s exact test, *p* = 0.014 ) and third (Fisher’s exact test, *p* = 0.018) learning tests (Figure 1D). Moreover, we found that the control bees did not show any response to the odor hexanol (odor B, CS −) during the three conditioning trials. The percentage of cadmium-exposed bees showing PER to hexanol was 3.2% and 1.6% in the first and second conditioning trial, respectively, and the cadmium-exposed bees showed no response to hexanol in the third conditioning trial (Figure S1). No significant differences were found between the two groups regarding the response to hexanol (Fisher’s exact test, first conditioning trial: *p* = 0.22; second conditioning trial: *p* = 0.47), suggesting the stability and specificity of the behavioral response in the two groups of bees.

### 3.3. Analysis of Brain Gene Expression Profiles

**Figure 2:**
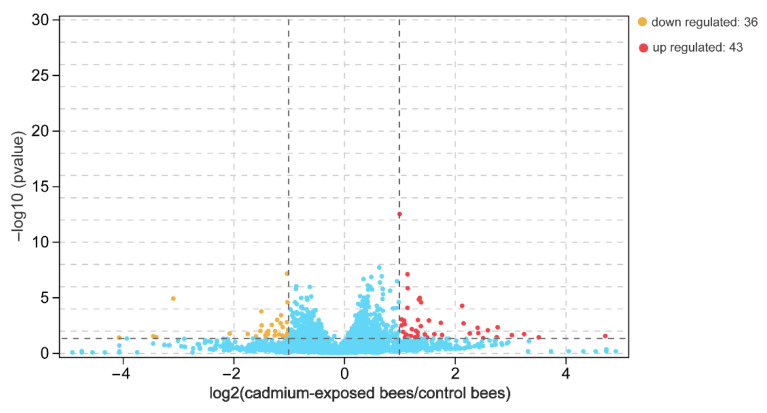
Differentially expressed genes in the brain between cadmium-exposed bees and control bees based on a *p*-value < 0.05 and an absolute fold-change > 2. Red and yellow indicate the up- and downregulated genes in cadmium-exposed bees, respectively; blue indicates no differentially expressed genes.

The number of paired-end clean reads mapped to the *Apis mellifera* L. reference genome was about 14.8 and 18.8 million for the sequencing libraries of the cadmium-exposed and control bees, respectively. The whole sequence reads have been deposited in the Genome Sequence Archive (GSA accession number CRA006897). We obtained a total of 10,488 genes, 596 of which were novel genes after mapping to the reference genome. A total of 79 significantly differentially expressed genes were obtained in the brain between the cadmium-exposed and the control bees based on the *p*-value and fold-change criteria (Table S2). Of these, 43 and 36 were upregulated and downregulated in the brains of the cadmium-exposed bees, respectively (Figure 2). Cd exposure downregulated genes encoding chemoreceptors and olfactory proteins that include odorant binding protein 14 (LOC677673) and odorant receptor Or1 (LOC100576462). The expression levels of the genes encoding the two proteins exhibited about an 8–10 fold decrease in the brain of the cadmium-exposed bees compared with that of the control bees. In addition, Cd exposure upregulated genes engaged in the response to oxidative stress, and these upregulated genes include cytochrome b5 (LOC726850), cytochrome P450 6a14 (LOC550965), tyrosine aminotransferase (LOC725204), and trehalase (LOC410484). The expression levels of the genes encoding these proteins exhibited about a 3–26 fold increase in the brains of the cadmium-exposed bees compared with that of the control bees.

### 3.4. GO and KEGG Enrichment Analysis

We listed the top ten significantly enriched GO terms associated with 13 unique differentially expressed genes (Table 1), and 2 and 11 genes were downregulated and upregulated in the brains of the cadmium-exposed bees, respectively. These significantly enriched GO terms include monooxygenase activity (GO:0004497), heme binding (GO:0020037), iron ion binding (GO:0005506), etc. All four genes (LOC100577883, LOC413908, LOC550965, and LOC551179), encoding different forms of cytochrome P450 enzymes involved in the monooxygenase activity, were significantly upregulated in the Cd-exposed bees compared to the control bees. Nine significant KEGG pathways associated with the 16 unique differentially expressed genes were identified in the cadmium-exposed bees, and 7 and 9 genes were downregulated and upregulated in the brains of the cadmium-exposed bees, respectively (Table 2). The pathways mainly include starch and sucrose metabolism, insect hormone biosynthesis, lysine biosynthesis, etc.

### 3.5. Validation of DEGs by qPCR

**Figure 3:**
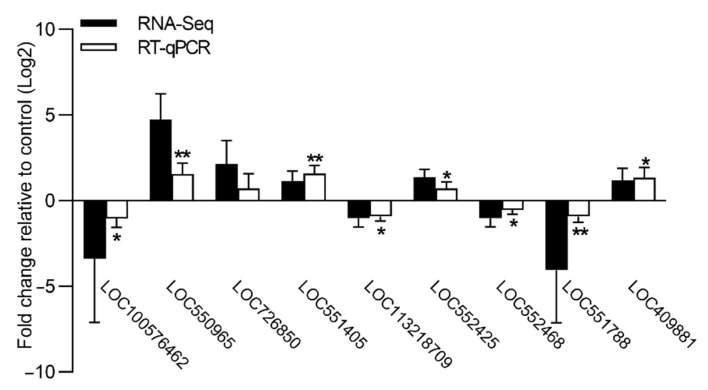
Validation of FPKM expression value (RNA-seq) for nine genes by RT-qPCR. The abscissa indicates the nine DEGs, and the ordinate indicates the relative expression levels of nine DEGs in the brains of the cadmium-exposed bees relative to the controls. The error bar indicates SD; the asterisk denotes significant differences (Student’s t-test, * *p* < 0.05; ** *p* < 0.01).

The relative expression levels of nine DEGs were further validated by qPCR (Figure 3). Consistent with the FPKM expression value generated by RNA-seq, the qPCR results revealed that all nine genes exhibited similar gene expression profiles in the brains of the cadmium-exposed bees relative to the controls. The genes encoding odorant receptor 1 (LOC100576462), inactive peptidyl-prolyl cis-trans isomerase shutdown (LOC113218709), MATH and LRR domain-containing protein PFE0570w-like (LOC552468), and katanin p60 ATPase-containing subunit A-like 2 (LOC551788) showed a significantly lower level of expression in the brains of the cadmium-exposed bees relative to the controls. The genes encoding cytochrome P450 6a14 (LOC550965), 4-nitrophenylphosphatase (LOC551405), glucose dehydrogenase (LOC552425), and myosin regulatory light chain 2 (LOC409881) showed a significantly higher level of expression in the brains of the cadmium-exposed bees relative to the controls. Therefore, the validation results of the nine DEGs confirmed the reliability of the transcriptome data.

## 4. Discussion

Burden et al. [38] measured the sucrose sensitivity of honey bees to five different doses of CdCl_2_ ranging from 0.001 μg/mL to 10 μg/mL and found that honey bees did not show any significant rejection response to sugar-water contaminated with CdCl_2_. However, the honey bees exhibited a reduction in consuming the sucrose containing not less than 1 ug/mL of CdCl_2_; although non-significant, there exists the possibility that sucrose containing more than 1 ug/mL of Cd may affect honey bee sucrose responsiveness that is essential for olfactory learning in honey bees [38,39]. Besides, honey bees fed diets containing 0.24 ug/mL of CdCl_2_ for up to 7 days had an alteration in the composition of the microbiome [40]. Therefore, a cadmium exposure dose of 0.25 ug/mL was used in our studies. Our data showed that there were no significant differences in both syrup consumption and survival rate between the cadmium-exposed and control bees, thereby further confirming that the Cd dose used in our studies is sublethal and exerts chronic toxic effects on honey bees. Di et al. [41] studied the acute toxic effects of CdCl_2_ on honey bees and found that honey bees fed Cd exhibited a significant reduction in syrup consumption compared to controls. The lowest Cd dose (26 mg/L) used in their studies was about 100-fold higher than the dose (0.25 μg/mL) used in our studies. A higher dose of Cd may cause malaise in honey bees, thus affecting the syrup consumption of honey bees [38]. In addition, it has been known that Cd exposure induces developmental defects including head deformities and causes a significant decrease in the total protein content in a broad range of vertebrate species [42,43,44,45]. Our studies showed that the head weight was significantly lower in cadmium-exposed bees when compared to controls, suggesting chronic Cd exposure may have adverse effects on the total protein content of the mandibular and hypopharyngeal glands in the heads of adult honey bees, thus resulting in a decrease in head weight in Cd-exposed bees. Previous studies reported that head size was significantly reduced in chronic lead-exposed honey bees that showed impaired olfactory learning performances [46]. Heavy metals including lead and Cd, therefore, have similar adverse effects on the cognitive abilities of honey bees. Odorant binding proteins (OBPs) play important roles in odor sensing in insects [47], and the interaction between OBPs and imidacloprid decreased the binding affinity of OBPs to a floral odor [48]. In addition, significantly downregulated expression levels of genes encoding OBPs were found in imidacloprid-treated bees exhibiting impaired olfactory learning [21]. A significant 8.5-fold decrease in the expression level of the gene encoding the odorant-binding protein 14 (Obp 14) was observed in the brains of cadmium-exposed bees, suggesting that the ability of odor recognition in cadmium-exposed bees may be weakened. It remains to be determined whether cadmium may exhibit a similar binding mode to the OBPs as neonicotinoid imidacloprid in the brains of honey bees. In addition, the expression level of *odorant receptor 1* (*Or1*) was downregulated more than 10-fold in the brains of the cadmium-exposed bees in comparison to the control bees. The odorant receptor was regarded as the primary receptor involved in the sensory detection of external chemical stimuli in insects [49]. Fibers originating from the *Or1*-expressing neurons in the pore plate sensilla of honey bees may enter the brain and innervate the glomeruli of the antennal lobe of the honey bee brain [50,51]. The dysregulation of *Or1* expression in the nerve fibers may have neurologically adverse effects on the transmission of odor information in the brains of honey bees; therefore, the decreased expression levels of both *Obp 14* and *Or1* probably resulted in the impaired olfactory learning of honey bees. However, further studies are required to elucidate the exact roles of *Obp 14* and *Or1* in the process of olfactory learning in honey bees. In addition, trehalase plays vital biological roles in recovery after abiotic stress through hydrolyzing trehalose in insects [52]. Cadmium exposure significantly increased the expression level of the gene encoding trehalase in the brain of honey bees, indicating that honey bees may need extra energy to combat cadmium stress. Diacylglycerol kinases (DGKs) play important roles in neuron physiology and behavior regulation, and the gene *Dgks* are highly expressed in the brain of mammals [53,54]. The knockout of certain *Dgk* isoforms resulted in substantially impaired cognitive functions in mice [54,55]. The expression level of the gene encoding the η isoform of DGK was significantly decreased in the brains of the cadmium-exposed bees. Like in mammals, the downregulation of *Dgk* may possibly affect the olfactory learning of honey bees.

Previous studies have demonstrated that cadmium exposure exerts oxidative stress in several tissues and causes neurotoxicity in organisms [10,56], and the brain is highly prone to oxidative damage [57]. Tyrosine aminotransferase (TATN) plays critical roles in oxidative stress response in organisms [58]. The gene encoding TATN was significantly upregulated in the brain of cadmium-exposed bees, suggesting that the upregulation of this gene may provide a protective function against oxidative stress induced by cadmium exposure. Furthermore, it is noteworthy that oxidative stress-related genes were all significantly upregulated in the brains of the cadmium-exposed bees, and these genes include cytochrome b5 (LOC726850), cytochrome P450 4aa1-like (LOC100577883), cytochrome P450 6A1 (LOC413908), cytochrome P450 6a14 (LOC550965) and cytochrome P450 15A1 (LOC551179). Cytochrome b5 (Cyt-b5) has been identified as a neuroprotective factor targeting oxidative damage in *Drosophila melanogaster* [59]. The expression level of *Cyt-b5* was significantly upregulated in the brains of the cadmium-exposed bees in comparison to the control bees, and the upregulation of *Cyt-b5* may play neuroprotective roles against oxidative stress induced by cadmium. Significantly increased levels of genes encoding cytochrome P450 (CYP) enzymes were observed in the brains of the cadmium-exposed bees, suggesting that the brain CYPs may play key roles in metabolizing the reactive oxygen species induced by Cd and influencing behavior and cognition in honey bees [60]. Further research is needed to investigate the interplay between the levels of brain CYPs and olfactory learning performances in honey bees. In addition, the starch and sucrose metabolism was shown to be involved in alleviating the toxic effects of Cd on poplars [61]. Two genes associated with the pathway were significantly upregulated in the cadmium-exposed bees, suggesting that this pathway may play a similar protective role for honey bees following Cd exposure. Additionally, previous studies demonstrated that hormone-induced antioxidant responses played important roles in the defense of abiotic and biotic stressors in insects [62]. Two genes associated with the insect hormone biosynthesis pathway were significantly upregulated in cadmium-exposed bees, indicating that the hormonal regulation of antioxidant response may also contribute to defense against Cd-induced oxidative stress in the brain of honey bees.

Taken together, our results indicated that Cd exposure exerted oxidative stress and decreased the gene expression levels of chemoreceptors in the brain of honey bees, which probably resulted in impaired olfactory learning in honey bees. Furthermore, increased expression levels of genes engaged in coping with oxidative stress may serve as a protector to combat oxidative stress and alleviate toxic effects caused by Cd exposure.

## Figures and Tables

**Table 1:** Enrichment analysis of top ten GO terms related to upregulated and downregulated genes in the brains of cadmium-exposed bees.

GO ID	GO Term	No. of Genes	*p* Value
GO:0020037	heme binding	6	1.61 × 10^−5^
GO:0046906	tetrapyrrole binding	6	1.73 × 10^−5^
GO:0048037	cofactor binding	9	0.000114
GO:0004497	monooxygenase activity	4	0.000895
GO:0005506	iron ion binding	4	0.001554
GO:0016705	oxidoreductase activity, acting on paired donors, with the incorporation of or reduction in molecular oxygen	4	0.002607
GO:0008483	transaminase activity	2	0.004673
GO:0016769	transferase activity, transferring nitrogenous groups	2	0.004673
GO:0042720	mitochondrial inner membrane peptidase complex	1	0.005238
GO:0004806	triglyceride lipase activity	2	0.005317

**Table 2:** Significantly enriched KEGG pathways related to upregulated and downregulated genes in the brains of cadmium-exposed bees.

Enriched KEGG Pathway	Upregulated Genes	Downregulated Genes	*p* Value
Starch and sucrose metabolism	LOC411257, LOC410484		0.00514
Insect hormone biosynthesis	LOC551179, LOC551405		0.00572
Lysine biosynthesis	LOC724239		0.0122
Metabolic pathways	LOC411257, LOC410484, LOC413678, LOC552425, LOC724239, LOC725026, LOC725204, LOC727237,	LOC412355, LOC725284, LOC727510	0.0137
Glycine, serine, and threonine metabolism	LOC413678, LOC552425		0.0198
Phenylalanine, tyrosine, and tryptophan biosynthesis	LOC725204,		0.0242
RNA transport		LOC100576378, LOC411217, LOC724659	0.0357
Phenylalanine metabolism	LOC725204		0.0479
Glycosphingolipid biosynthesis—globo and isoglobo series		LOC727510	0.0479

## Data Availability

The data presented in this study are available on request from the corresponding author.

## Supplementary Materials

The following supporting information can be downloaded at: https://www.mdpi.com/article/10.3390/insects13110988/s1, Figure S1: The percentage of bees showing PER to CS -; Table S1: The primer sequences used in qRT-PCR; Table S2. A list of differentially expressed genes in the brains of cadmium-exposed and control bees.
