# Supplementary figures and images for: Chronic Cadmium Exposure Induces Impaired Olfactory Learning and Altered Brain Gene Expression in Honey Bees (Apis mellifera)
Source: Insects. 2022 Oct 27;13(11):988. doi: 10.3390/insects13110988 (PMC9696575; doi:10.3390/insects13110988)

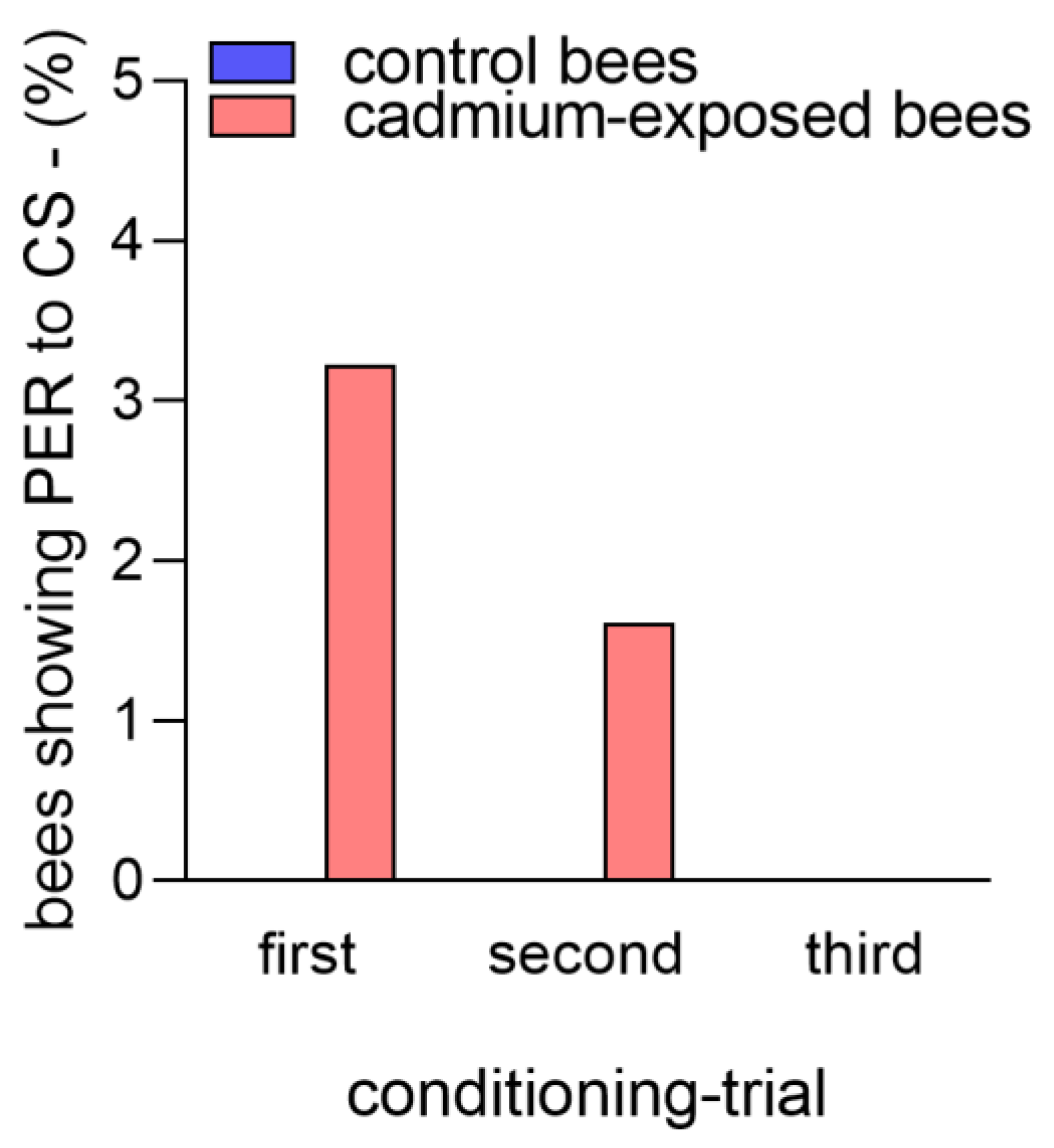

Supplement: Supplementary file 1 [file insects-13-00988-s001.zip › Figure S1. The percentage of bees showing PER to CS -.tif]
